# Nigrostriatal dopaminergic depletion increases static orofacial allodynia

**DOI:** 10.1186/s10194-016-0607-z

**Published:** 2016-02-17

**Authors:** Wisam Dieb, Omar Ouachikh, Sofia Alves, Yves Boucher, Franck Durif, Aziz Hafidi

**Affiliations:** UFR Odontologie, Université Paris Diderot, Paris, France; Centre de Psychiatrie et Neurosciences, INSERM U894, Paris, France; Clermont Université, Université d’Auvergne, EA7280, Clermont-Ferrand, France; CHU Clermont-Ferrand, Service de Neurologie, 63000 Clermont-Ferrand, France

**Keywords:** Parkinson disease, Dopamine, Pain, Substantia nigra, Chronic constriction injury, Neuropathic pain, Infraorbitary nerve

## Abstract

**Background:**

This study investigated mesencephalic dopamine depletion effects on static mechanical allodynia (SMA) elicited by chronic constriction of the infraorbitary nerve (CCI-IoN).

**Methods:**

Dopamine depletion (6-OHDA administration into the medial forebrain bundle) effects on CCI-IoN-induced SMA were explored using behavioral (nocifensive behavior score upon non-noxious stimuli using von Frey filament), pharmacological (bromocriptine injections) and immunohistochemical (PKCγ and pERK1/2) techniques.

**Results:**

The central dopamine depletion increased significantly the SMA score. Intraperitoneal and intracisternal injections of bromocriptine alleviated the allodynic behavior observed in both CCI-IoN and CCI-IoN + 6-OHDA animal groups. At the cellular level, dopamine depletion induced a significant increase in PKCγ expression in the medullary dorsal horn (MDH) in rat with CCI-IoN + 6-OHDA when compared to sham animals (CCI-IoN only). Similarly, after static non-noxious stimuli, the expression of pain marker proteins pERK1/2 within the MDH revealed significantly a higher number of positive cells in CCI-IoN + 6-OHDA rats when compared to the CCI-IoN group.

**Conclusion:**

This study demonstrates that nigrostriatal dopamine depletion exacerbates the neuropathic pain resulting from CCI-IoN. This effect is probably due to an action through descending pain inhibitory systems which increased pain sensitization at the MDH level. It demonstrates also an analgesic effect elicited by D2R activation at the segmental level.

**Electronic supplementary material:**

The online version of this article (doi:10.1186/s10194-016-0607-z) contains supplementary material, which is available to authorized users.

## Background

Painful traumatic trigeminal neuropathy (PTTN) following peripheral nerve trauma is a disabling condition clinically characterized by spontaneous and evoked pain mainly experienced as burning and/or shooting pain [1]. It results from dysfunctions of the somatosensory system [2] and remains a therapeutic challenge since the current treatment options are unsatisfactory [3]. The physiopathogeny of PTTN points to both peripheral mechanisms involving neuro-glio-immuno vascular alterations mediated by chemokines/cytokines release and central mechanisms involving both alterations of ascending pathways and descending controls [4–7].

Dopamine has been proposed as playing a key role in chronic orofacial pain [8]. Nigrostriatal dopamine depletion is associated with increased pain sensitivity and is implicated in pain in different pathologies such as Parkinson’s disease, restless leg syndrome, fibromyalgia, burning mouth syndrome and atypical facial pain [9–13]. Conversely, striatal administration of dopamine 2 receptor (D2R) agonists has an anti-nociceptive effect mediated by the rostro-ventromedial medulla (RVM) [14]. Similar results showed that striatal inhibition of nociceptive responses evoked in the trigeminal system [15–17] and chronic oro-facial pain conditions in humans were associated with the alteration of the nigrostriatal dopaminergic system [9, 10]. Recent reports showed that bilateral or unilateral nigrostriatal dopaminergic lesions induce dynamic and static mechanical allodynia in the oro-facial region [16, 18]. Since the effects of dopamine depletion on the development of PTTN have not been explored, the present study aimed at investigating the effects of nigrostriatal lesions in animals with a peripheral nerve injury: chronic constriction injury of the Infra Orbitary Nerve (chronic constriction of the infraorbitary nerve: CCI-IoN) model [19]. This model has the advantage of using the trigeminal nerve for pain related studies [20]. Bilateral nigrostriatal chemical lesions were performed by stereotaxic injection of the 6-OHDA toxin into medial forebrain bundle (MFB) in rats with CCI-IoN. In these animals, pain behavior (nocifensive, mechanical allodynia), expression of PKCγ [(protein involved in pain chronicity) [21, 22]] and pERK1/2 (proteins expressed upon noxious stimuli in the spinal/medullary dorsal horn [23, 24] were assessed with or without dopamine receptor (DR) agonists [bromocriptine (D2R), SKF81297 (D1R)] treatments.

## Methods

### Animals

Adult male Sprague–Dawley rats (*N* = 112, 275-325 g) from Charles River (L’Arbresle, France) were maintained in a controlled environment (lights on 07:00–19:00, 22 °C) with *ad libitum* access to food and water. The experiments followed the ethical guidelines of the International Association for the Study of Pain, the European Community Council directive of 24 November 1986 (86/609/EEC) and the Animal Ethics Committee of the University of Auvergne.

### 6-OHDA lesion

After anesthesia (Ketamine 60 mg/kg, xylazine, 10 mg/kg), rats were placed in a stereotaxic frame (David Kopf Instrument, CA, USA) and the MFB were injected bilaterally with 6-OHDA (0.5 μL/min) dissolved in a vehicle solution (0.02 % ascorbate saline) at a concentration of 3 μg/μL (Sigma-Aldrich, France) in two deposits (2.25 and 2.85 μg, respectively) at the following coordinates: anterior (A) −4.0; lateral (L) ± 0.8; ventral (V) -8.0; tooth bar at +3.4 and A −4.4; L ± 1.2; V −7.8; tooth bar at −2.4 [25]. To preserve adrenergic neurons from 6-OHDA toxicity, animals received desipramine (25 mg/kg, i.p., Sigma-Aldrich, France) 30 min prior to the toxin injection; sham-lesioned rats received only the vehicle at the same coordinates.

### CCI-IoN surgery

CCI-IoN was performed following an established surgical procedure [5, 19]. Briefly, animals were anesthetized using chloral hydrate (400 mg/kg i.p.) and an incision of approximately 1 cm long was made along the gingivobuccal margin, begun just proximal to the first upper molar. About 0.5 cm of the IoN was freed of adhering tissue and two ligatures (4–0 chromic guts) separated by a 1–2 mm interval were tied loosely around it using 4–0 chromic gut. The sham operation was identical except that the nerve was not ligated.

### Behavioral testing and analysis

The rats were adapted to the observation field (24 × 35 × 18 cm) and for 30 min each day for 9 days prior to the beginning of behavioral testing. During this period, the experimenter reached into the cage to apply von Frey (2 g) stimulus on the animals’ faces. For each behavioral testing, the rats were placed in the observation field for a 30 min period. Stimulation was carried out when the rat was in a sniffing/no locomotion state: with four paws placed on the ground, neither moving nor freezing. The stimulus was applied every 3 min onto the vibrissal pad (IoN territory). Each series of stimulation consisted of 5 von Frey filament (2 g) applications every 5 s alternating on each side of the face. This stimulus is non-noxious.

Behavioral responses were quantified by a blind-experimenter according to the method of [19]: (1) detection, the rats turn heads toward stimulus; (2) withdrawal reaction (the rats turn head away); (3) escape/attack, the rats avoid further contact with the stimulus, or attack the filament; (4) asymmetric grooming, the rats display an uninterrupted series of at least three wash strokes directed at the stimulated area. An absence of response corresponded to a zero score. A mean score value was then calculated for each series of stimulations. All the rats were subjected to 13 sessions of behavioral testing at different time points: before surgery (day 1) and after surgery, on weeks 1, 2, 3, 4, 5, 6, 7, 8, 9, 10, 11 and 12.

### Immunohistochemistry

A day after behavioral experiments, rats were deeply anesthesized with urethane (1.5 g/kg i.p), the vibrissal pads were ipsilaterally stimulated for 2 min by a von Frey filament 2 g (60 stimuli delivered, 0.5 Hz), and three minutes later, the rats were perfused transcardially with warm (37 °C) heparinized saline (25 IU heparin/ml) followed by cold (10 °C) phosphate-buffered solution (0.1 M, pH 7.6) containing 4 % paraformaldehyde and 0.03 % picric acid. The brains were placed in 30 % sucrose and 0.05 % sodium azide solution overnight at 4 °C. Brainstem coronal sections (30 μm) were cut on a freezing microtome and collected in 0.05 M Tris-buffered saline (TBS).

Free-floating sections were placed in 1 % normal goat serum for 1 h before overnight incubation at room temperature in primary antibody solutions (mouse anti-pERK1/2 [1:1000, Cell Signaling Technologies], and rabbit anti-PKCγ [1:4000, Sigma-Aldrich and Santa Cruz]. The corresponding secondary antibodies (1:400 for goat anti-mouse Cy3, 1:200 for goat anti-rabbit Cy2) were incubated at room temperature for 3 h. All antibodies were diluted in TBS containing 0.25 % bovine serum albumin and 0.3 % Triton X-100. The sections were finally rinsed in TBS, mounted onto gelatin-coated slides, dehydrated in alcohol, cleared in xylene, and cover-slipped with distyrene-plasticizer-xylene. The specificity of the immunostaining was assessed by omitting primary antibodies, which resulted in the absence of signal.

Immunostaining was analyzed using as motorized Zeiss Axioplan 2 microscope equipped with a Hamamatsu C4742-95 digital camera driven by MetaMorph® 5.4 software. In each rat, image acquisition and fluorescent signal quantification were performed from 7 different sections, each taken at a given rostrocaudal plane within the MDH (from 0 to −2160 μm at 360 μm intervals). Brainstem sections were categorized according to their approximate rostrocaudal location from the MDH subnucleus interpolaris junction. pERK1/2 positive cells were counted and data were expressed as the sum of the total number of labeled cells counted from all sections analyzed in each animal [16]. PKCγ staining was quantified as previously reported [16]. Briefly, PKCγ staining was quantified within lamina IIi and the number of positive cells was counted in lamina III. Tyrosine hydroxylase (TH) immunolabelling was performed (anti-TH primary antibody; Millipore, France) as described above. The quantification procedure of the 6-OHDA lesion impact on the SNc was reported previously [25].

### Drugs and administration

Two weeks after the 6-OHDA injection, the animals were briefly (<3 min) anesthetized with 2 % halothane using a mask and received for intracisternal administration bromocriptine (7 μg/kg dissolved in 5 μl vehicle; Sigma-Aldrich, France) or the vehicle alone (5 μl of 0.9 % saline) according to our previous results [25]. For i.p. injection we used bromocriptine (1 mg/kg) and SKF81297 (3 mg/kg dissolved in 0.9 % saline; Sigma-Aldrich, France) concentrations [25]. Following a recovery period (<2 min), the rats were placed in the observation field for 40-min period-test by a blind-experimenter.

### Statistical analysis

The results are expressed as mean ± SD. Statistical analysis was performed using Student’s t-test, or a one-way analysis of variance (ANOVA) followed by a post hoc Student Newman-Keuls test or a one-way Repeated Measures (RM) ANOVA followed by a post hoc Student-Newman-Keuls test. The level of significance was set at P < 0.05.

## Results

### Dopamine depletion in the substantia nigra

As shown in our previous study [25], 6-OHDA injections resulted in a considerable decrease of TH staining in the SNc of CCI-IoN + 6-OHDA when compared to CCI-IoN + saline animals (Additional file 1: Figure S1A and B). Cell count revealed a significant (*p* < 0.001, ***) decrease in TH positive cells (70 % neuronal loss) in SNc of CCI-IoN + 6-OHDA rats (Additional file 1: Figure S1C). The impact of unilateral or bilateral depletion mesencephalic midbrain depletion on SMA has been studied previously [18].

### Dopamine depletion increases static mechanical allodynia (SMA) resulting from CCI-IoN

**Fig. 1:**
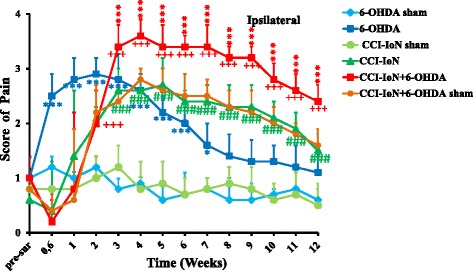
Time course of static mechanical allodynia (SMA) in the ipsilateral side to the injury (CCI-IoN) of 6 animal groups (each *n* = 8): CCI-IoN and its sham; 6-OHDA and its sham; and 6-OHDA + CCI-IoN and its sham. CCI-IoN animals showed a significant increase in the nocifensive score at post-surgery week 2 when compared to CCI-IoN-sham. The SMA score reached its maximum around the 4th week post-surgery and decreased slowly through the post-surgery observation period until week 12. SMA occurred rapidly within 3–4 days after 6-OHDA injection. This related SMA stayed significant when compared to sham for 7 weeks after the lesion. The highest nocifensive score was observed in the 6-OHDA + CCI-IoN group in comparison to all other groups. Despite the slight decrease observed along the 12 weeks post-surgery, the SMA score in this group remained significantly increased compared to all other groups. Comparison between CCI-IoN + 6-OHDA and CCI-IoN and 6-OHDA. Comparison between CCI-IoN and CCI-IoN sham.  Comparison between CCI-IoN + 6-OHDA and CCI-IoN + 6-OHDA sham.  Comparison between 6-OHDA and 6-OHDA sham. The bars represent standard deviation. *P < 0.05; **P < 0.01;***p < 0,001

The time course of SMA appearance (Fig. 1), resulting from the CCI-IoN, was similar to our previous report [16]. Briefly, 1 week post-surgery, significant SMA score (CCI-IoN) was obtained when compared to sham and reached its highest score (*p* < 0.001, ***) 3 weeks post-surgery. This SMA was significant during 12 weeks post-injury.

The time course of 6-OHDA induced SMA appearance was similar to our previous report [7]. The SMA appeared relatively earlier after 6-OHDA injection than after CCI-IoN (Fig. 1) and stayed significant in comparison to 6-OHDA-sham during the first 6 weeks after the 6-OHDA lesion.

The CCI-IoN + 6-OHDA rats showed the highest significant (*p* < 0.001, ***) SMA score within the ipsilateral side (Fig. 1) to the CCI-IoN injury in comparison to all other animal groups. This score was highly significant along the experiment duration. Similar results were obtained in the contralateral side, although with lower scores (data not shown).

### Bromocriptine administration decreases the SMA

**Fig. 2:**
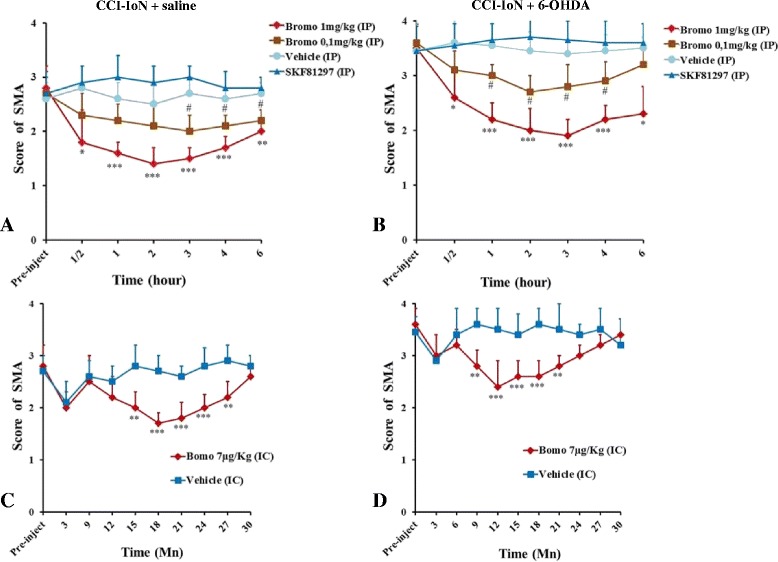
Effects of intraperitoneal (ip) (**a**, **b**) and intracisternal (ic) (**c**, **d**) administration of bromocriptine, SKF81297 and vehicle on nocifensive behaviors (SMA) in the ipsilateral side of CCI-IoN + saline (**a** & **c**) and CCI-IoN + 6-OHDA (B&D). Ip administration (**a**) of 1 mg/Kg of bromocriptine induces a significant antinociceptive effect in 6-OHDA-lesioned animal (*n* = 8) when compared to the vehicle group *n* = 8). This antinociceptive effect occurred 20 min after bromocriptine injection and lasted for up to 6 h. When administered ic (**b**), bromocriptine (1 μg/Kg, *n* = 5) decreases significantly the SMA score in comparison to the vehicle group (*n* = 5). The effect of bromocriptine was observed 20 min after injection and this effect lasted for about 15 min. No significant difference in SMA scores was observed when bromocriptine was injected intracisternally 90 min after the ip administration of sulpiride (**c**). Error bars = standard deviation. *P < 0.05; **P < 0.01; ***p < 0.001. *: comparison between bromo 1 mg/kg (IP) and vehicle. #: comparison between bromo 0.1 mg/kg (IP) and vehicle

Intraperitoneal administration of bromocriptine induced a significant, dose dependent (0.1 mg and 1 mg/Kg) decrease in pain scores in CCI-IoN group when compared to sham (Fig. 2a) and its effect lasted for 6 h. The highest dose induced the highest score decrease, (*P* < 0.01, **). As a positive control SKF8129 (DR1 agonist) was used. Its intraperitoneal administration induced a non-significant increase in the SMA score when compared to sham (saline-injected).

Intracisternal administration of bromocriptine (Fig. 2b) decreased significantly the SMA score when compared to sham (saline-injected). Bromocriptine effect lasted for 20 min.

Intraperitoneal administration of bromocriptine (Fig. 2c) induced a significant dose dependent decrease in SMA score in CCI-IoN + 6-OHDA lesioned group compared to that of sham. Its effect lasted for 6 h. SKF81297 administration increased the allodynic score, although this score was not significant when compared to sham. Intracisternal administration of Bromocriptine (Fig. 2d) decreased significantly the SMA score compared to that of sham (saline-injected rats) and its effect lasted for 30 min.

#### PKCγ expression in the medullary dorsal horn

**Fig. 3:**
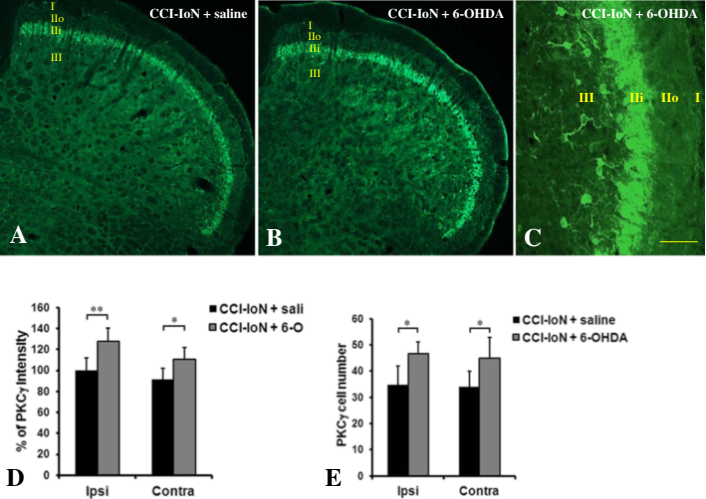
Expression of PKCγ in saline-CCI-IoN (**a**) and 6-OHDA-CCI-IoN (**b**) animals. Intense PKCγ labeling was observed within the MDH in lamina IIi cells and in scattered cells of lamina III in both animal groups, but the highest staining signal was observed in the 6-OHDA- group (**b**). **c** A high magnification showing the intense label within lamina IIi and in cells within lamina III. PKCγ staining intensity analysis (**d**) showed a significant increase in 6-OHDA injected animals within lamina IIi in both ipsi (*P* < 0.01) and contralateral (*P* < 0.05) sides of the MDH. PKCγ positive cell count within lamina III (**e**) showed a significant increase (*P* < 0.05) in cell number for 6-OHDA-CCI-IoN compared to saline-CCI-IoN animals. Scale bar represents 100 μm in A-B and 30 μm in C

The general pattern of PKCγ staining was similar in both CCI-IoN (Fig. 3a) and CCI-IoN + 6-OHDA (Fig. 3b) groups. PKCγ staining (Fig. 3c) was highly observed within lamina IIi and in scattered cells within lamina III [16, 22]. PKCγ staining intensity quantification in lamina IIi (Fig. 3d) was significantly higher in both ipsilateral (*P* > .001, **) and contralateral (*P* > .05,*) MDH sides of CCI-IoN + 6-OHDA when compared to CCI-IoN rats. There was no significant PKCγ staining difference between ipsi- and contralateral sides in lamina IIi. Cell count of PKCγ positive cells in lamina III (Fig. 3e) was significantly increased in CCI-IoN + 6-OHDA when compared to CCI-IoN groups. There was no significant cell count difference between ipsilateral and contralateral sides.

#### Increased pERK1/2 expression in the MDH by 6-OHDA

**Fig. 4:**
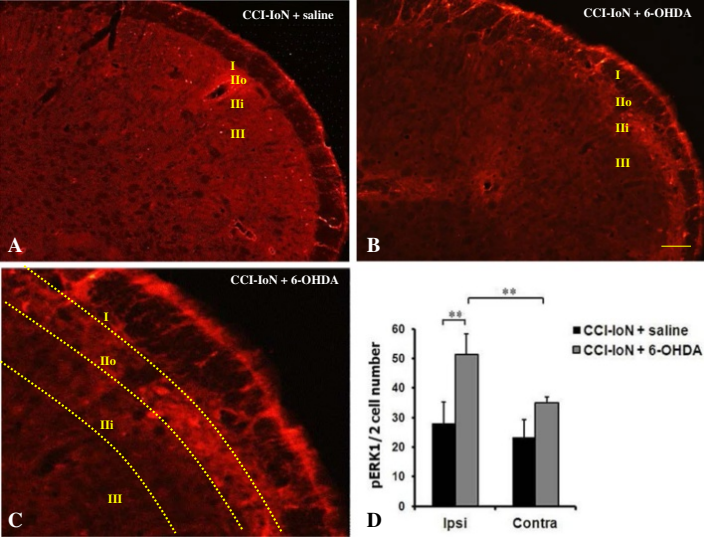
Expression of pERK1/2 in saline-CCI-IoN (**a**) and 6-OHDA-CCI-IoN animals (**b**-**c**). pERK1/2 positive cells were observed within superficial laminae of the MDH in both groups. At high magnification the pERK1/2 positive cells were observed mostly in laminae IIo and I as delimited (yellow dots) in the figure (**c**). Cell count (**d**) of pERK1/2 positive cells showed a significant increase (*P* < 0.01, **) in 6-OHDA vs saline injected animals (*n* = 5 each group) in the ipsilateral MDH and a non-significant increase in the contralateral side. A significant increase in the number of pERK1/2 positive cells was observed in the ipsilateral side of 6-OHDA injected animals compared to the contralateral side. Scale bar represents 100 μm in A-B and 40 μm in C

pERK1/2 staining was observed in cells located principally within superficial lamina II and I of the MDH (Fig. 4a-c). At higher magnification the pERK1/2 labeling was observed in cells located mostly in laminae I and IIo (Fig. 4c). Cell count of pERK1/2 positive cells was significantly (*P* < .01, **) higher in CCI-IoN + 6-OHDA (Fig 4d) when compared to CCI-IoN injured rats in the ipsilateral side to the injury (stimulated side). There was no significant difference in the number of pERK1/2 cells between CCI-IoN and CCI-IoN + 6-OHDA in the contralateral side. However there was a significant (*P* > .01, **) cell count difference between ipsi-and contralateral sides in both groups.

### PKCγ and pERK1/2 are distinct cell subtypes

**Fig. 5:**
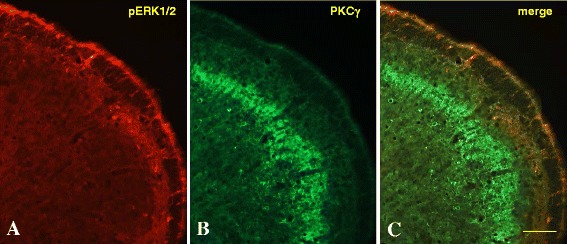
Double labeling using pERK1/2 (**a**) and PKCγ (**b**) antibodies in CCI-IoN + 6-OHDA rats. No co-localization (**c**) between markers was observed in MDH cells demonstating distinct cell subtypes for these markers. The bar represents 100 μm in A-C

pERK1/2 (Fig. 5a) and PKCγ (Fig. 5b) double labeling revealed no co-localization of these markers in both CCI-IoN and CCI-IoN + 6-OHDA MDH (Fig. 5c).

A summary illustration of the present results is presented in the following table (+low, +++high).

**Table:** 

	CCI-IoN	6-OHDA	CCI-IoN + 6-OHDA
SMA	++	+	+++
PKCγ	++	+	+++
pERK	++	+	+++

## Discussion

The main results of this study are: 1) Mesencephalic dopamine depletion augmented significantly the pain caused by CCI-IoN. 2) Bromocriptine administrations (intraperitoneal and intracisternal) attenuated SMA in both CCI-IoN and CCI-IoN + 6-OHDA animals. 3) Central dopamine depletion increased significantly PKCγ and pERK1/2 expressions in the MDH of CCI-IoN + 6-OHDA when compared to CCI-IoN group.

This study shows a synergistic or additional effect of central dopamine depletion and CCI-IoN on SMA in oro-facial territories. The dopamine depletion increased the SMA caused by CCI-IoN. This is in agreement with our previous studies demonstrating the induction of dynamic and static [16, 18] mechanical allodynia in the trigeminal system upon midbrain dopamine depletion. Bromocriptine attenuated CCI-IoN-related SMA in a dose dependent manner. These results highlights an MDH local action of bromocriptine by the activation of D2R since SKF81297 (D1R agonist) had no significant effect on pain behavior. These data are in accordance with previous results showing a direct inhibition of superficial spinal dorsal horn neurons by activation of D2R [26, 27].

At the molecular level, central depletion of dopamine increased synergistically the pre-existing expressions of PKCγ and pERK1/2 within MDH. PKCγ is known to be a key molecule for the onset of pain chronicity [21]. Its expression increases after CCI-IoN confirming previous studies [22, 24, 28]. Our result suggests that the lesion of the dopaminergic nigrostriatal system increased the SMA by acting on PKCγ cells through descending pain inhibitory system. PKCγ cells are known to activate a secondary cell subtype within MDH superficial laminae which expressed pERK1/2 [28] and the specific PKCγ inhibition decreased both the number of pERK1/2 cells and the related neuropathic pain behavior [24]. This highlights the essential role of PKCγ cells in inducing allodynia. PKCγ cells constitute a revolving door that induces allodynia through peripheral (CCI-IoN) or central lesions (nigrostriatal system). Moreover, bromocriptine administration has been shown in the same neuropathic model to decrease PKCγ expression levels in the MDH [16], thus suggesting that dopamine might act directly on PKCγ cells either by direct inhibition or indirectly through D2Rs present at excitatory presynaptic site at the level of PKCγ cells. In support of the latter, D2Rs have been detected post-synaptically on second-order neurons [29].

The implication of basal ganglia, in the processing of noxious somatosensory information is well documented [30]. Activation of the dopaminergic nigrostriatal system leads to a general anti-nociceptive effect [31], while its alteration enhances sensitivity to noxious stimuli [32, 33]. The anti-nociceptive dopamine effect is achieved through D2R receptors [14, 16, 31, 33].

In agreement with these data, the use of bromocriptine in the present study demonstrated also the involvement of D2R receptor in the anti-nociceptive effect resulting from both central dopamine depletion and CCI-IoN.

To date no direct nigral projections to the MDH is documented. Therefore, the increase of SMA is probably due to the indirect modulation of the pain descending modulatory system through the periaqueductal grey matter (PAG) [34]. The administration of apomorphine (a dopamine receptor agonist) into the PAG promotes anti-nociception [35]. GABA-ergic projections from SNc, substantia nigra reticula, ventral tegmental area and amygdala to the PAG have been described [36, 37]. Dopamine depletion in these structures may decrease GABA transmission at PAG level, thereby increasing the influence of descending facilitatory pain pathways on the MDH through the RVM. The latter represents the final common pain modulatory system [34]. Stimulation of striatal D2R suppressed nociceptive neuropathic pain through RVM modulation and activation of D2R and 5-HT receptors at the dorsal spinal horn [14]. The meso-limbic and meso-cortical dopamine projections can also participate to the increase of pain caused by CCI-IoN since 6-OHDA injection has been demonstrated to induce dopaminergic cell degeneration in the VTA [16, 18, 33]. Alternatively the segmental action of bromocriptine (intracisternal injection) may act via dopaminergic descending pathway which arise from the hypothalamus region A11 [38]. Other brain structures which receive mesencephalic dopamine innervation could also modulate nociception at the MDH level [39].

Our data supported the implication of the mesencephalic dopamine system in the PD, orofacial (burning mouth syndrome, atypical facial pain) and other pain-related pathologies (restless leg, fibromyalgia) (9.10.11.12, 13). It is worth to note that in PD, patients experimented pain during the Off-period (absence of dopamine replacement therapy) which highlighted the general role for dopamine in pain process. Thus central dopamine may have a general inhibitory action on pain directly by dopaminergic projections to descending pain control or indirectly through dopamine projections to target nuclei and its depletion may causes a general pain increase.

## Conclusion

In conclusion the present study demonstrated a synergic effect of CCI-IoN and central dopamine depletion in neuropathic pain. The nigrostriatal dopamine increased allodynic behavior through D2R at segmental PKCγ. D2R agonists might be used as analgesic mechanism for trigeminal allodynia.

## Additional file

Additional file 1: Figure S1. Tyrosine Hydroxylase (TH) immunostaining in saline + CCI-IoN (A) and 6-OHDA + CCI-IoN (B) animals (*n* = 8) revealed a drastic decrease in the staining intensity mainly observed in the substantia nigra pars compacta (SNc). The cell count (C) demonstrated a significant (*p* < 0.001, ***) decrease in TH positive cells in the SNc of 6-OHDA rats when compared to shams. Scale bar = 170 μm in A-B. Error bar = standard deviation in C. ****p* < 0.001. (DOC 274 kb)
