# Supplementary figures and images for: Nigrostriatal dopaminergic depletion increases static orofacial allodynia
Source: J Headache Pain. 2016 Feb 17;17:11. doi: 10.1186/s10194-016-0607-z (PMC4757596; doi:10.1186/s10194-016-0607-z)

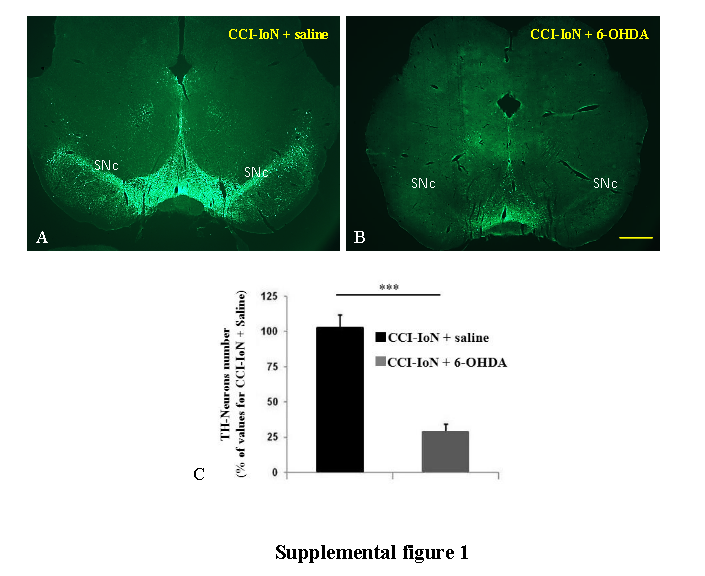

Supplement: Additional file 1: Figure S1. — Tyrosine Hydroxylase (TH) immunostaining in saline + CCI-IoN (A) and 6-OHDA + CCI-IoN (B) animals (n = 8) revealed a drastic decrease in the staining intensity mainly observed in the substantia nigra pars compacta (SNc). The cell count (C) demonstrated a significant (p < 0.001, ***) decrease in TH positive cells in the SNc of 6-OHDA rats when compared to shams. Scale bar = 170 μm in A-B. Error bar = standard deviation in C. ***p < 0.001. (DOC 274 kb) [file 10194_2016_607_MOESM1_ESM.doc]
